# Age-Correlated Gene Expression in Normal and Neurodegenerative Human Brain Tissues

**DOI:** 10.1371/journal.pone.0013098

**Published:** 2010-09-29

**Authors:** Kajia Cao, Alice S. Chen-Plotkin, Joshua B. Plotkin, Li-San Wang

**Affiliations:** 1 Department of Pathology and Laboratory Medicine, University of Pennsylvania, Philadelphia, Pennsylvania, United States of America; 2 Department of Neurology, University of Pennsylvania, Philadelphia, Pennsylvania, United States of America; 3 Institute on Aging, University of Pennsylvania, Philadelphia, Pennsylvania, United States of America; 4 Center for Neurodegenerative Diseases, University of Pennsylvania, Philadelphia, Pennsylvania, United States of America; 5 Department of Biology, University of Pennsylvania, Philadelphia, Pennsylvania, United States of America; 6 Penn Center for Bioinformatics, University of Pennsylvania, Philadelphia, Pennsylvania, United States of America; Tokyo Medical and Dental University, Japan

## Abstract

**Background:**

Human brain aging has received special attention in part because of the elevated risks of neurodegenerative disorders such as Alzheimer's disease in seniors. Recent technological advances enable us to investigate whether similar mechanisms underlie aging and neurodegeneration, by quantifying the similarities and differences in their genome-wide gene expression profiles.

**Principal Findings:**

We have developed a computational method for assessing an individual's “physiological brain age” by comparing global mRNA expression datasets across a range of normal human brain samples. Application of this method to brains samples from select regions in two diseases – Alzheimer's disease (AD, superior frontal gyrus), frontotemporal lobar degeneration (FTLD, in rostral aspect of frontal cortex ∼BA10) – showed that while control cohorts exhibited no significant difference between physiological and chronological ages, FTLD and AD exhibited prematurely aged expression profiles.

**Conclusions:**

This study establishes a quantitative scale for measuring premature aging in neurodegenerative disease cohorts, and it identifies specific physiological mechanisms common to aging and some forms of neurodegeneration. In addition, accelerated expression profiles associated with AD and FTLD suggest some common mechanisms underlying the risk of developing these diseases.

## Introduction

### Human brain aging and neurodegenerative diseases

Among human tissues, the brain in normal aging and in neurodegenerative disease has received special attention. This interest stems largely from the observation that as humans age most develop some degree of cognitive decline. While slight, differences in performance in tasks of visual and verbal memory [1], [2], abstraction [3], and naming and verbal fluency [4] exist between aged individuals and their younger counterparts. Moreover, measurable differences in cognitive performance are seen in both cross-sectional and longitudinal-design studies [5]. In daily life, these differences are too small and too common to be considered pathological. In some aging individuals, however, pathological decline in cognitive function in the form of a dementing neurodegenerative illness does develop.

Alzheimer's disease (AD) is the most well known of these dementing neurodegenerative diseases. Affecting at least 4.5 million Americans [6], AD causes decline in memory as well as other aspects of cognition [7]. Although less common, another neurodegenerative cause of dementia is frontotemporal lobar degeneration (FTLD). Clinically, patients with FTLD exhibit progressive decline in behavior, executive function (e.g. ability to make decisions), and language.

In the case of AD, specific genes and proteins have been implicated in pathogenesis (reviewed in [8], [9]). For example, important genetic risk factors for AD include APOE genotype, while rare mutations in PS1, PS2, and APP can cause primarily familial forms of disease [10]. In addition, specific proteins (beta-amyloid, tau) certainly play a role in disease pathogenesis, aggregating in different forms in diseased vs. normal tissue [11], [12]. While substantial evidence implicates these genes and proteins in AD pathogenesis, their precise functions in the pathological cascade that leads to AD is still a subject of considerable debate.

Regarding FTLD, a substantial proportion (precise number?) of cases are attributable to mutations in either the gene for progranulin (*GRN*), which cause disease through a haploinsufficiency mechanism (reviewed in [13]) or the MAPT gene [14]. Other cases of FTLD remain enigmatic, but, as in AD, the aggregation of specific proteins, such as the recently discovered 43kD TAR DNA-binding protein (TDP-43, [15]), appears to play a role (reviewed in [16], [17], [18], [19]). For both diseases, the clinical course is progressive and ultimately fatal, and neuropathological examination reveals extensive neurodegeneration. While the core area of neurodegeneration differs among the diseases (hippocampal formation in AD, frontal and temporal lobes in FTLD), both diseases affect the frontal cortex.

The risk of developing a neurodegenerative disease increases with age. For example, in one US study, the estimated annual incidence of Alzheimer's Disease (AD) increased from 0.6% in individuals aged 65–69 years, to 2% in those aged 75–80 years, to 8.4% in those aged 85 years and older [6]. Not all neurodegenerative diseases occur with greater frequency as individuals age, however. In FTLD, for instance, prevalence may be higher in individuals aged 60–69 years than in those aged 70–79 years [20]. When evaluated worldwide without reference to cause, however, rates of dementia consistently increase with age despite variability between regions of the world and between developed and developing countries [21].

These findings beg the question of whether “normal” and “pathological” cognitive decline are really on a spectrum, with one's position on that spectrum shifting with age. Put another way, one might ask whether there are underlying processes in common between aging and neurodegenerative disease. Common mechanisms in aging and neurodegeneration certainly seem plausible. Indeed, mechanisms such as mitochondrial dysfunction and DNA damage have been implicated in both aging and neurodegeneration (reviewed in [22]). More generally, aging has long been understood to be accompanied by cumulative insults to cells that eventually lead to their degeneration and demise.

### Profiling gene expression in aging and neurodegenerative diseases

With the advent of technology allowing for genome-wide surveys of gene expression, studies of aging have become feasible at the molecular level. Using gene expression microarrays, genes associated with the aging process have been identified [23], [24], [25]. Moreover, these genes have been used as age biomarkers to establish the physiological age of organisms [26], [27], detect tissue-specific aging differences [24], [28], [29], [30] and study the biology of aging-related diseases [31], [32].

These advances also make it possible to address the question of whether aging and neurodegeneration are mechanistically similar in a novel way. Clinical and pathological descriptors are fairly downstream phenotypes for a process, be it neurodegeneration or aging. In contrast, global mRNA expression profiles are very upstream phenotypes that hint at specific biological mechanisms. Indeed, we and others have shown that extensive transcriptional changes occur in brain tissue from patients with AD [31], [33], and FTLD [16], [34], with specific molecular pathways implicated in both diseases (reviewed in [16]). Although these microarray studies have attempted to control for the effect of age on gene expression, a question that remains unanswered (and largely unasked) is that of how similar the transcriptional changes that occur with aging are to the transcriptional changes that occur with specific neurodegenerative diseases.

### Outline of the paper

In this study, we developed a method using global gene expression data to accurately determine the age of a normal brain sample. We then employed this method to ask whether samples from patients with neurodegenerative diseases look “older” at a molecular level than their age-matched non-diseased counterparts. We examined neurodegenerative diseases that increase in incidence with age (*e.g.* AD), and those in which the relationship between age and disease incidence is unclear (*e.g.* FTLD). We found that both neurodegenerative diseases, in specific regions of the brain, show characteristics of accelerated aging.

## Results

### Age prediction using brain gene expression

We used three reference datasets of gene expression in normal human brain to train and test our age predictor using datasets D1∼D3 in Table 1. As described in the Methods section, error in age prediction was estimated using data from 80% of the subjects to predict ages in the remaining 20%. We found that age prediction by global gene expression was accurate within 10.5 to 16 years of the actual age of the subject, with variation in the error depending on the brain region used (Table 2). When we randomly permuted the ages of the individuals in each brain dataset (1000 permutations performed), no more than 0.5% of the permutations had age prediction errors that were less than our observed error of 10.5∼16 years. We also found that the difference of actual and predicted median age on the same group of subjects (between 5 and 6 subjects in the 20% partition) by cross validation is between 5.41 and 8.83 years. The decrease in error is proportional with the square root of sample size minus 1 and is similar to the behavior of standard deviation as one moves from estimating individuals to estimating population behavior. We estimate the error will be between 2.24 and 3.41 years when estimating the median age of 23 AD samples. Therefore, our age predictor performed significantly better than would be expected by random chance, and can be used to study the population accurately.

**Table 1:** Microarray data sets used in this paper and corresponding human brain regions.

Brain regions	Normal	FTLD	AD
Rostral aspect of frontal	**D1:** 29 [24]	**D4:** [16] *	**D5:** [33]
cortex (∼BA10)	Age: 26∼95; F: 11; M: 18	GRN+: 6	AD : 23
	GEO#: GDS707	Age: 62∼79; F: 3; M: 3	Age: 68∼90; F: 10; M: 13
		GRN−: 10	Ctrl: 11
		Age: 48∼77; F: 6; M: 4	Age: 63∼102; F: 4; M: 7
		Ctrl: 8	GEO#: GSE5281
		Age: 47∼92; F: 3; M: 5	(Superior frontal gyrus covers
		GEO#: GSE13162	both BA9 and BA10)
Dorsolateral prefrontal	**D2:** 29 [28] *		
cortex (BA9)	Age: 25∼79; F: 7; M: 22		
BA47	**D3:** 27 [28] *		
	Age: 28∼77; F: 6; M: 21		

*obtained from the authors directly.

**Table 2:** Significance of the error by obtaining 1,000 randomized cross-validation errors with age information randomly shuffled; the significance of the prediction error is the fraction of the 1,000 randomized errors lower than the actual cross-validation error.

	D1 (BA10)	D2 (BA9)	D3 (BA47)
Error in age prediction	16.09±7.69	11.15±5.62	10.49±6.92
(five-fold cross validation)			
Difference of median of age	7.92	8.83	5.41
(with actual age)			
Median of difference of age	6.86	8.20	6.21
(with actual age)			
Permutation test P values	0	0.005	0.002

### Region-specific correlations between age and gene expression levels in human frontal cortex

**Figure 1:**
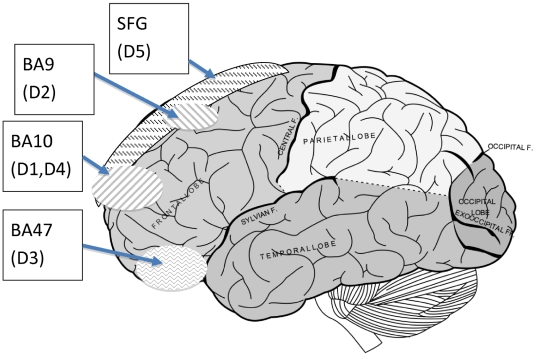
Microarray data sets used in this paper and corresponding human brain regions. Regions and abbreviations: SFG, superior frontal gyrus; BA10, rostral aspect of frontal cortex; BA9, dorsolateral prefrontal cortex; and BA47, orbital prefrontal cortex. See Table 1 for detailed information of correspondence between brain regions and data sets used in this study. The brain illustration is downloaded from wikipedia.org, a reproduction based on the 1918 edition Gray's Anatomy. The image is in the public domain; see Licensing on http://en.wikipedia.org/wiki/File:Gray728.svg.

**Figure 2:**
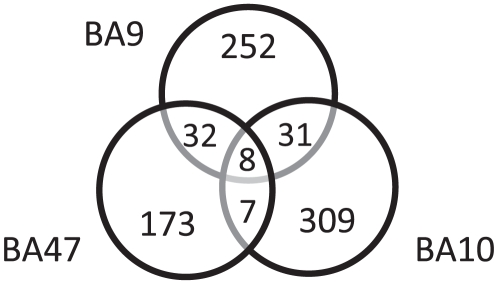
Venn diagram of three human brain regions. Age-correlated genes (p≤0.01) in three human brain regions are very different. Shown are the numbers of genes with age-correlated expression in each brain region.

As described in the Methods section, we tested our age predictor using three independent normal human brain datasets. These profiled gene expression in three different areas of the frontal cortex: the rostral part of the frontal cortex (roughly BA10, D1) [24], dorsolateral prefrontal cortex (BA9, D2), and orbital prefrontal cortex (BA47, D3) [28] (Figure 1). Subjects in each dataset were between 20 and 95 years old, with individuals in every decade. We ran linear regressions on the three datasets and applied a nominal p value cut-off at 0.01 to obtain three age-correlated gene lists. We examined the overlaps among the three datasets (D1∼D3), and found that the identities of the age-correlated genes in the three regions were very different (Figure 2). Out of over 810 genes with age-correlated gene expression in at least one Brodmann Area, only 40 genes showed age-correlated gene expression in both BA9 and BA47, and only 39 genes showed age-correlated gene expression in both BA9 and BA10. BA47 and BA10 had the least overlap, with only 15 genes showing age-correlated gene expression in both areas.

For each of the three datasets, we analyzed the biological significance of the age-correlated genes using Gene Set Enrichment Analysis (GSEA) [35]. We focused on Category 5 (Gene Ontology, GO) and analyzed genes positively correlated with aging (*i.e.* greater age = greater expression) and genes negatively correlated with aging (*i.e.* lesser age = greater expression) separately. As shown in Table S1, we found that age-correlated genes in dataset D2 (BA9) were functionally more similar to D3 (BA47). Of note, many genes negatively correlated with age appeared to be involved in mitochondrial function.

### Application of age predictor to neurodegenerative disorders

Having demonstrated that estimation of chronological age using global brain gene expression is possible in normal individuals, we next turned our attention to samples from individuals with neurodegenerative diseases. We examined two different neurodegenerative diseases: AD, a disease highly associated with aging; and FTLD, a disease possibly associated with aging. For the analysis of microarray datasets pertaining to these two neurodegenerative disorders, we matched the brain region of reference data (used to build the predictor) and target data (control and patient gene expression profiles) (Figure 1). We did this because of the regional differences in age-correlated gene expression described in the previous section. See Table 1 for pairing of reference and target datasets.

**Figure 3:**
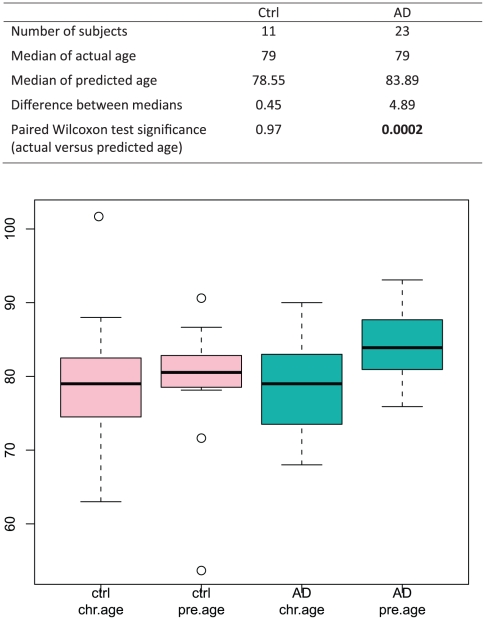
Comparison between actual and predicted ages of controls and AD patients. We compared observed and predicted ages for controls and AD patients. While the difference between observed and predicted age was not significant for controls, AD patient samples had a significantly older predicted age compared to the actual observed age. (Top) Numbers of subjects, medians of ages, and P-values for Wilcoxon test are shown. (Bottom) Box and whiskers plots of observed and predicted ages for controls and AD patients. Box represents median (bar) and interquartile range, while whiskers represent range of all values excepting outliers (shown as open circles). Reference data set used for training our age predictor was D2 (BA9), and genes used for age prediction were selected using a nominal p-value cut-off of 0.005.

Applying our age predictor to AD, we found that brain samples from AD patients had an “older” expression profile than control samples of the same chronological age. The median chronological age for the AD patients (23 individuals between 68 and 90 years, D5 in Table 1) was 79 years. However, by global gene expression trained on region-matched normal controls (D2 in Table 1), the median predicted age for the AD cohort was 83.89 years. In contrast, neurologically normal control samples from the same microarray study (11 individuals between 63 and 102 years, D5 in Table 1) showed minimal differences in median chronological age (79 years) and age as predicted by gene expression (78.55 years). The difference between chronological and predicted ages in AD was highly significant (*p* = 0.0002), whereas the difference in chronological and predicted ages in normal controls was not (*p* = 0.97) (Figure 3). Taken together, these data indicate that AD superior frontal gyrus samples show a prematurely aged global gene expression profile. Global gene expression trained on BA10 (D1 in Table 1) and similar prediction on AD patients also led to the same conclusion (Figure S1).

Having seen an “older” expression profile in AD brains compared to normal controls, we next evaluated FTLD, one of the most common causes of dementia after AD. Specifically, we considered the form of FTLD with underlying TDP-43 pathology (FTLD-TDP), which is the most common neuropathological substrate of the clinical entity FTLD [18], [36]. A significant proportion of FTLD-TDP is caused by mutations in the *GRN* gene [13], and we have previously demonstrated that FTLD-TDP with *GRN* mutations has a frontal cortex gene expression profile distinct from FTLD-TDP without *GRN* mutations [16]. We therefore analyzed the two subgroups of FTLD-TDP, cases with and without *GRN* mutations, separately. As with AD, we matched brain regions used in our training set and test set (D1 and D4, respectively, in Table 1) and normalized across datasets using the expression of housekeeping genes.

**Figure 4:**
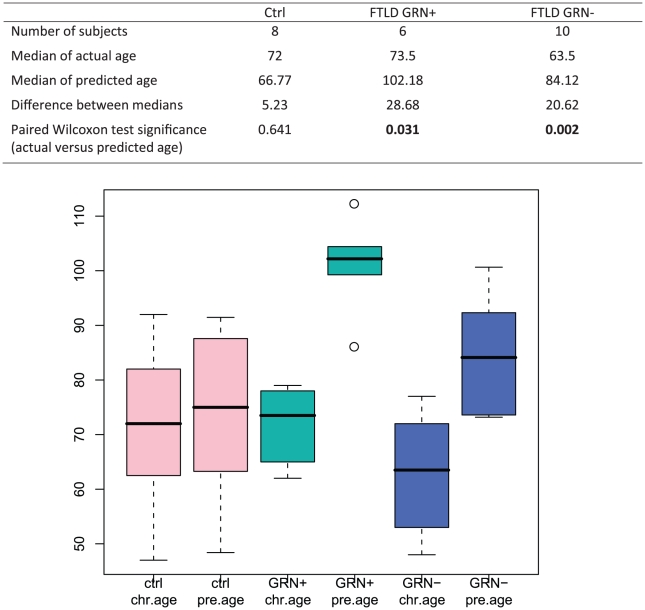
Comparison between actual and predicted ages of controls and FTLD-TDP patients. We compared observed and predicted ages for controls and FTLD-TDP patients with (GRN+) and without (GRN−) *GRN* mutations. While the difference between observed and predicted age was not significant for controls, FTLD-TDP patient samples had significantly older predicted ages compared to actual observed ages, regardless of *GRN* mutation status. (Top) Numbers of subjects, medians of ages, and P-values for Wilcoxon test are shown. (Bottom) Box and whiskers plots of observed and predicted ages for controls and FTLD-TDP patients. Box represents median (bar) and interquartile range, while whiskers represent range of all values excepting outliers (shown as open circles). Reference data set used for training our age predictor was D1 (BA10), and genes used for age prediction were selected using a nominal p-value cut-off of 0.005.

Despite small sample sizes (6 FTLD-TDP patients with *GRN* mutations, 10 FTLD-TDP patients without *GRN* mutations), we found that both subgroups had a significantly “older” gene expression profile than neurologically normal controls of the same chronological age. Specifically, FTLD-TDP patients with *GRN* mutations had a median chronological age of 73.5 years but a median predicted age of 102.18 years as estimated by frontal cortex global gene expression (p = 0.031). FTLD-TDP patients without *GRN* mutations had similarly “aged” brain gene expression, with a median chronological age of 63.5 years but a median predicted age of 84.12 years (p = 0.002). In contrast, neurologically normal controls from the same dataset (8 individuals ranging from 47 to 92 years of age, D4 in Table 1) had a median chronological age (72 years) that was not significantly different from median predicted age (66.77 years, p = 0.641) (Figure 4). Thus, global gene expression in both FTLD-TDP and AD brain appears markedly “older” than the actual chronological age of patient samples.

### Overlap between age-correlated genes and differentially expressed genes in neurodegenerative disorders

We next evaluated the overlap of aging-correlated genes (nominal p<0.005) and AD- or FTLD-TDP-associated genes, (nominal p<0.001) in the same or similar regions of the brain (Table S2). As would be predicted by our findings of a “prematurely aged” gene expression profile in AD, we found a statistically significant overlap of age-correlated genes and AD-associated genes (p = 3.34×10^−4^ by Fisher's exact test). Similarly, in FTLD-TDP, where we also found a “prematurely aged” gene expression profile, we observed a statistically significant overlap of age-correlated genes and FTLD-TDP-associated genes in both FTLD-TDP patients with *GRN* mutations (p = 2.02×10^−6^ by Fisher's exact test) and FTLD-TDP patients without *GRN* mutations (p = 1.21×10^−4^ by Fisher's exact test).

### Towards the discovery of common mechanisms in aging and FTLD

Our finding of prematurely aged gene expression in FTLD-TDP and AD implies that aging and these neurodegenerative diseases have mechanisms in common and leads to the question of what these common mechanisms might be.

Having demonstrated in the previous section that a significant overlap exists between genes associated with aging and with FTLD-TDP, we evaluated the identity of those genes showing differential expression in both aging and FTLD-TDP. The rationale for doing so was to find genes and pathways shared between physiologic aging and pathophysiologic mechanisms in FTLD-TDP. Because of the large number of genes with altered expression in FTLD-TDP, we used relatively stringent statistical (p<0.001) and fold-change (FC>2) cut-offs to identify genes robustly associated with disease [16]. 17 genes (Table S3) showed differential expression in both aging and FTLD-TDP. For 16 of these genes, the direction of age-associated gene expression (e.g. higher in aged individuals vs. younger ones) was the same as the direction of FTLD-TDP-associated gene expression (e.g. higher in FTLD-TDP patients vs. controls) (Table S3).

Among these genes, HSPA2 encodes a heat-shock protein of the 70kD family, which operates as a molecular chaperone in response to cellular stress. LAMP2 encodes a protein that functions specifically in the maintenance of lysosomes and more generally in the regulation of autophagy. Autophagy (reviewed in [37], [38]) and heat-shock proteins (reviewed in [39]) have been implicated in both aging and neurodegeneration, corroborating our general result that some neurodegenerative diseases have mechanisms in common with aging.

## Discussion

In this paper, we developed a computational method for assessing an individual's “physiological brain age” based on global mRNA expression. We applied our method to five human brain microarray datasets and found that global gene expression can be used to predict the chronological age of a normal brain sample. We further found that age-correlated gene expression differs among different regions of brain. Finally, we applied our age-prediction method to demonstrate that the neurodegenerative diseases FTLD-TDP and AD exhibit prematurely aged gene expression profiles in specific brain regions.

Among normal samples, our model was able to predict the chronological age of a sample within approximately 11 years in two datasets (D2 and D3). Others have found a similar degree of error using global gene expression to predict age [28] in datasets obtained under the same experimental conditions on the same microarray platforms. Our methodology therefore allows for a comparable degree of accuracy in age prediction under the much noisier conditions introduced by independently obtained datasets. This advantage is important when considering applications of cross-dataset analyses.

It is worth noting here that the difference in age predicted by global gene expression and chronological age for a given sample may not simply be due to error in prediction methodology. That is, an individual's physiological age – defined as that age most accurately representing the sum of insults to cells, tissues, and organs that make up the individual – may not coincide exactly with his or her chronological age. One would expect gene expression profiles to reflect this concept of physiological age and, as such, to only approximate the chronological age even in the case of a perfect method. This point was elegantly illustrated in a recent study of global gene expression at different time points across the lifespan of the nematode worm [40], where behavioral phenotypes were used as proxies for physiological age in addition to straightforward comparison with chronological age.

Our finding that different regions of human brain exhibit different patterns of age-correlated gene expression corroborates work by others [41] demonstrating prominent differences in age-related gene expression between samples from the superior frontal gyrus and precentral gyrus. Such a finding is not surprising given the different connections and functions of even neighboring brain regions, but it does mean that attention must be paid to regional differences when interpreting mRNA expression profiling data.

Using our age prediction method, we showed that AD and FTLD-TDP patient brains exhibit prematurely aged gene expression profiles. Such a finding supports the intuitive notion that aging and at least these two neurodegenerative diseases have mechanisms in common. Although not surprising, our finding is nonetheless important for several reasons. First, we have established in a quantitative way on a common scale the degree to which various neurodegenerative diseases resemble aging (*e.g.* AD “adds 5 years” to a brain sample). Second, we can identify specific physiologic/pathophysiologic mechanisms common to both aging and various neurodegenerative diseases. An example of such an application is the identification of autophagy and heat-shock response genes with altered expression in both aging and FTLD-TDP. The fact that a substantial body of literature already exists linking these two biological pathways to both aging and neurodegeneration lends validity to our approach; other genes and pathways identified in a similar manner may provide avenues for future research.

We considered the possibility that our finding of a “prematurely aged” global gene expression signature is simply an artifact of neuronal loss. While we cannot completely exclude this possibility, the fact that we observe such different age-correlated genes in adjacent areas of frontal cortex (which are similarly affected in terms of neuronal loss) argues that our findings are unlikely to be due to neuronal loss alone.

Our finding of prematurely aged gene expression in FTLD-TDP and AD suggests the possible commonalities between aging and these neurodegenerative diseases, and supports the notion that transcriptome profiling can be an informative approach for investigating these commonalities, when larger datasets for normal brain aging become available. It remains to be shown how much the overlap corresponds to any common mechanism between normal aging and neurodegenerative disorders or common responses without etiological implications. However, recent advances in expression quantitative trait linkage (eQTL) [42], [43], [44] may eventually provide a causal link connecting some susceptible loci and changes in gene expression.

## Materials and Methods

### Data preparation

Microarray datasets used in this paper were either generated by us as previously described (Chen-Plotkin et al. 2008), downloaded from GEO (http://www.ncbi.nlm.nih.gov/geo/index.cgi), or obtained from the authors directly. Table 1 summarizes the data used in this paper. For all datasets, the GCRMA package [45] for R/Bioconductor [46] was used to generate log-2 expression levels for probeset IDs from the original .cel files. Ages for healthy individuals used in this study ranged from 20 to 95 years. For the purposes of this study, individuals with psychiatric diagnoses from one dataset [28] were classified with normal controls, as they had previously been shown not to differ in age-related gene expression from individuals without psychiatric diagnoses.

### Predicting age using microarray experiments

We used linear regression to compute the significance of a correlation between age and the expression level of a gene, adjusting for the effect of gender. This approach assumes a linear relationship between age and log-2 expression level:
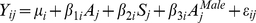
(1)Here *Y_ij_* is the log-2 gene expression level of probe set *i* in sample *j*, *A_j_* is the age for individual *j*, *S_j_* is 0 if individual *j* is female, 1 if he is male. *A_j_^Male^* is the age of individual *j* if *S_j_* = 1; it is 0 otherwise (included to test for interaction between age and gender). The coefficients *b_1i_*, *b_2i_*, and *b_3i_* are regression coefficients reflecting the rate of change in gene expression with respect to age alone, gender alone, and age-gender interaction effects, respectively.

The model was computed on normal individuals in different brain regions (Table 1). To minimize the interaction of gender in age prediction, we filtered out any genes that have *p*≥0.05 for *b_2i_* and *b_3i_*. Then genes significantly correlated with age (*p*≤0.005 for *b_1i_* were used in the predictor to estimate the physiological age of control subjects and subjects with neurodegenerative disease in the corresponding brain regions D1, D2, D3. We used five-fold cross validation to estimate the error of our age predictor; see Supplemental Methods S1 for more details.

In order to apply our age predictor across diverse microarray experiments, we needed to address two issues: microarray platform differences and baseline differences attributable to variations in experimental technique. To address the former, we used the best-match probeset ID tables provided by Affymetrix (https://www.affymetrix.com/support/technical/comparison_spreadsheets.affx) to match probeset IDs on different human genome microarrays used in this paper. For the latter, we assumed that the difference between two microarray experiments is a constant offset. We adjusted this baseline difference by estimating the difference between the expression levels of housekeeping genes common to the two datasets. 575 established housekeeping genes [47] were used in this calibration. We found that the difference between median predicted age and median actual age were 3.01 years and 2.99 years, and none were significant by Wilcoxon's test (Figure S2). This suggests that experimental baseline differences between microarray studies can be ignored. See Supplemental Methods S1 for details and validation of the calibration procedure.

### Application of age-predictor to disease datasets

For each neurodegenerative disease studied, we trained our age predictor on a reference dataset assaying gene expression in normal controls from the same brain region used in the target dataset (microarray dataset consisting of diseased individuals and their neurologically normal controls). We then applied the age predictor to two different test sets: the normal controls within the target dataset and the diseased individuals within the target dataset. We calculated the median predicted age for each test set and compared it to the median chronological age for the same test set. We then evaluated the significance of the difference between predicted ages and chronological ages in the test set using a paired Wilcoxon test. We examined the overlap between differentially expressed genes in neurodegenerative disorders age-correlated genes in region-matched normal brain aging. The significance of overlap was evaluated using Fisher's exact test. The lists of genes both differentially expressed in neurodegenerative diseases (AD, FTLD-TDP) and correlated with age can be found in Table S3, Table S4 and Table S5.

## Supporting Information

Figure S1 (0.14 MB PDF)

Figure S2 (0.14 MB PDF)

Table S1 (0.26 MB PDF)

Table S2 (0.20 MB PDF)

Table S3 (0.18 MB PDF)

Table S4 (0.18 MB PDF)

Table S5 (0.19 MB PDF)

Supplemental Methods S1 (0.27 MB PDF)
