# Supplementary material for: Age-Correlated Gene Expression in Normal and Neurodegenerative Human Brain Tissues
Source: PLoS One. 2010 Sep 29;5(9):e13098. doi: 10.1371/journal.pone.0013098 (PMC2947518; doi:10.1371/journal.pone.0013098)

Figure S1. Comparison between actual and predicted ages of controls and AD patients using reference data set D1.

We compared observed and predicted ages for controls and AD patients. While the difference between observed and predicted age was not significant for controls, AD patient samples had a significantly older predicted age compared to the actual observed age. (Top) Numbers of subjects, medians of ages, and P-values for Wilcoxon test are shown. (Bottom) Box and whiskers plots of observed and predicted ages for controls and AD patients. Box represents median (bar) and interquartile range, while whiskers represent range of all values excepting outliers (shown as open circles). Reference data set used for training our age predictor was D1 (BA10), and genes used for age prediction were selected using a nominal p-value cut-off of 0.005.

	Ctrl	AD
Number of subjects	11	23
Median of actual age	79	79
Median of predicted age	91.15	99.80
Difference between medians	12.15	20.80
Paired Wilcoxon test significance (actual versus predicted age)	0.24	**2.38×10^{-7}**

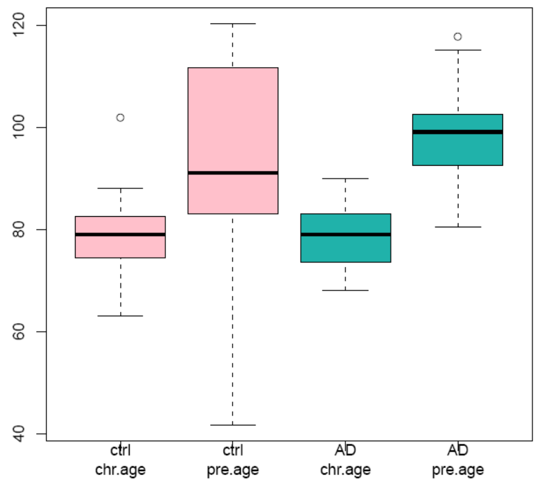

Supplement: Figure S1 — (0.14 MB PDF) [file pone.0013098.s001.pdf]
