# Supplementary material for: Age-Correlated Gene Expression in Normal and Neurodegenerative Human Brain Tissues
Source: PLoS One. 2010 Sep 29;5(9):e13098. doi: 10.1371/journal.pone.0013098 (PMC2947518; doi:10.1371/journal.pone.0013098)

Figure S2. Comparison between actual and predicted ages before and after microarray platform calibration.

(Top) Comparison by Wilcoxon test and (bottom) box-whisker plot of actual and predicted ages before and after microarray platform calibration. The calibration procedure is independent of age as it is only based on the difference in house-keeping gene expression levels across platforms. Box represents median (bar) and interquartile range. The reference data set used in calibration is D1 (BA10) and the target data set is the controls from D4. Age predictors are selected using p cutoff at 0.005.

	Actual Age	Predicted Age	
		Before calibration	After calibration
Median	72	68.99	74.99
Difference of median of age (with actual age)	-	3.01	2.99
Paired Wilcoxon test significance	-	0.742	0.641

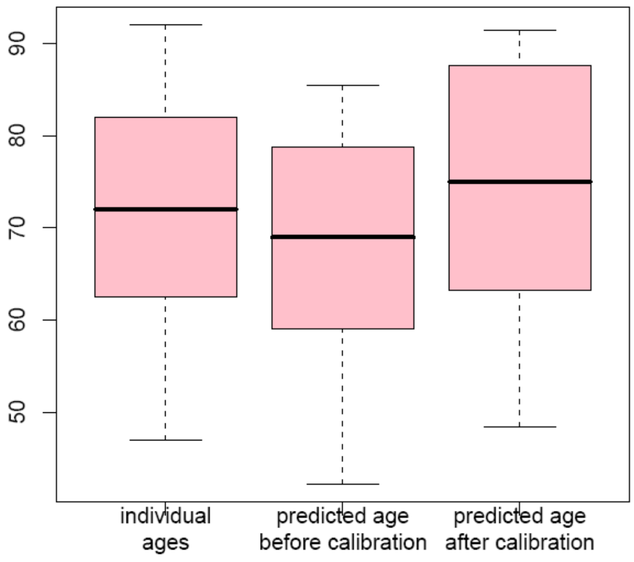

Supplement: Figure S2 — (0.14 MB PDF) [file pone.0013098.s002.pdf]
