# Supplementary material for: Age-Correlated Gene Expression in Normal and Neurodegenerative Human Brain Tissues
Source: PLoS One. 2010 Sep 29;5(9):e13098. doi: 10.1371/journal.pone.0013098 (PMC2947518; doi:10.1371/journal.pone.0013098)
Supplement: Table S1 — (0.26 MB PDF) [file pone.0013098.s003.pdf]

Table S1. Gene Set Enrichment Analysis for age-correlated genes in four normal brain aging datasets.

GO ID	Description	GSEA Significance		
		BA9 (D2)	BA10 (D1)	BA47 (D3)
Positively Correlated				
GO:0005576	extracellular region	**0.00E+00**	-	**0.00E+00**
GO:0044421	extracellular region part	**0.00E+00**	9.98E-01	**0.00E+00**
GO:0005615	extracellular space	**0.00E+00**	-	**4.00E-04**
GO:0006952	defense response	**0.00E+00**	1.00E+00	**0.00E+00**
GO:0009605	response to external stimulus	**0.00E+00**	1.00E+00	**0.00E+00**
GO:0050801	ion homeostasis	**0.00E+00**	-	**8.01E-04**
GO:0007186	G-protein coupled receptor protein signaling pathway	**0.00E+00**	-	**0.00E+00**
GO:0048513	organ development	**0.00E+00**	-	**4.00E-04**
GO:0006968	cellular defense response	**0.00E+00**	7.53E-01	**8.08E-04**
GO:0001584	rhodopsin-like receptor activity	**0.00E+00**	-	**0.00E+00**
GO:0004930	G-protein coupled receptor activity	**0.00E+00**	-	**0.00E+00**
GO:0004888	transmembrane receptor activity	**0.00E+00**	-	**0.00E+00**
GO:0065008	regulation of biological quality	**0.00E+00**	-	1.60E-03
GO:0008284	positive regulation of cell proliferation	**8.00E-04**	9.87E-01	2.83E-01
GO:0009888	tissue development	**4.00E-04**	-	1.60E-03
GO:0030003	cation homeostasis	**0.00E+00**	-	3.21E-03
GO:0045595	regulation of cell differentiation	**0.00E+00**	9.97E-01	3.82E-01
GO:0007600	sensory perception	**0.00E+00**	-	5.76E-02
GO:0042592	homeostatic process	**0.00E+00**	-	1.00E-02
GO:0019725	cell homeostasis	**0.00E+00**	8.85E-01	6.80E-03
GO:0007187	G-protein signaling, coupled to cyclic nucleotide second messenger	**4.00E-04**	-	2.13E-02
GO:0008283	cell proliferation	**0.00E+00**	-	3.52E-02
GO:0048878	chemical homeostasis	**0.00E+00**	6.33E-01	1.60E-03
GO:0007507	heart development	**8.05E-04**	-	2.46E-03
GO:0055080	The regulation of the levels, transport, and metabolism of cations	**4.00E-04**	-	4.40E-03
GO:0019935	cyclic-nucleotide-mediated signaling	**4.00E-04**	-	1.76E-02
GO:0003008	organ system process	**0.00E+00**	-	4.00E-03
GO:0007398	ectoderm development	**0.00E+00**	-	1.16E-02
GO:0004497	monooxygenase activity	**4.03E-04**	-	2.56E-01
GO:0001653	peptide receptor activity	**0.00E+00**	-	7.82E-02
GO:0005198	structural molecule activity	**0.00E+00**	1.43E-03	1.76E-02
GO:0005125	cytokine activity	**4.00E-04**	-	1.20E-03
GO:0044428	nuclear part	9.92E-01	**8.85E-04**	1.00E+00
GO:0043228	non-membrane-bound organelle	-	**4.38E-04**	-
GO:0043232	intracellular non-membrane-bound organelle	-	**4.34E-04**	-
GO:0006396	RNA processing	9.88E-01	**0.00E+00**	1.00E+00

GO:0006397	mRNA processing	9.56E-01	**0.00E+00**	-
GO:0008380	RNA splicing	9.68E-01	**0.00E+00**	-
GO:0003735	structural constituent of ribosome	1.22E-01	**0.00E+00**	9.83E-02
GO:0003723	RNA binding	8.22E-01	**0.00E+00**	9.86E-01
GO:0004222	metalloendopeptidase activity	3.26E-02	-	**0.00E+00**
GO:0007610	behavior	2.80E-03	7.43E-01	**4.00E-04**
GO:0007626	locomotory behavior	2.52E-02	-	**4.01E-04**
GO:0007267	cell-cell signaling	1.32E-02	-	**0.00E+00**
GO:0006955	immune response	2.40E-02	9.34E-01	**0.00E+00**
GO:0019932	second-messenger-mediated signaling	1.20E-03	-	**0.00E+00**
GO:0002376	immune system process	4.00E-03	9.57E-01	**0.00E+00**
GO:0045449	regulation of transcription	7.20E-03	3.30E-02	**8.00E-04**
GO:0008236	serine-type peptidase activity	3.29E-02	-	**0.00E+00**
GO:0004175	endopeptidase activity	3.84E-02	-	**0.00E+00**
GO:0017171	serine hydrolase activity	2.93E-02	-	**8.14E-04**
GO:0008233	peptidase activity	4.16E-02	-	**0.00E+00**
GO:0004252	serine-type endopeptidase activity	1.45E-02	-	**0.00E+00**
Negatively Correlated				
GO:0008135	translation factor activity, nucleic acid binding	**0.00E+00**	-	7.27E-02
GO:0019866	organelle inner membrane	-	1.38E-01	**0.00E+00**
GO:0044429	mitochondrial part	-	5.02E-01	**0.00E+00**
GO:0030529	ribonucleoprotein complex	-	-	**0.00E+00**
GO:0031966	mitochondrial membrane	-	4.48E-01	**0.00E+00**
GO:0031980	mitochondrial lumen	6.67E-01	-	**0.00E+00**
GO:0005740	mitochondrial envelope	-	3.39E-01	**0.00E+00**
GO:0044455	mitochondrial membrane part	-	6.61E-02	**0.00E+00**
GO:0007166	cell surface receptor linked signal transduction	-	**0.00E+00**	-
GO:0007267	cell-cell signaling	-	**0.00E+00**	-
GO:0051345	positive regulation of hydrolase activity	-	**0.00E+00**	-
GO:0007188	G-protein signaling, coupled to cAMP nucleotide second messenger	-	**0.00E+00**	-
GO:0019932	second-messenger-mediated signaling	-	**0.00E+00**	-
GO:0007187	G-protein signaling, coupled to cyclic nucleotide second messenger	-	**0.00E+00**	-
GO:0007186	G-protein coupled receptor protein signaling pathway	-	**0.00E+00**	-
GO:0019935	cyclic-nucleotide-mediated signaling	-	**0.00E+00**	-
GO:0003008	organ system process	-	**0.00E+00**	-
GO:0042165	neurotransmitter binding	-	**0.00E+00**	-
GO:0030594	neurotransmitter receptor activity	-	**0.00E+00**	-
GO:0001584	rhodopsin-like receptor activity	-	**0.00E+00**	-
GO:0004872	receptor activity	-	**0.00E+00**	-
GO:0004888	transmembrane receptor activity	-	**0.00E+00**	-
GO:0022857	Catalysis of the transfer of a substance from one side of a membrane to the other	-	**0.00E+00**	-

GO:0015075	ion transporter activity	-	**0.00E+00**	-
GO:0022892	Enables the directed movement of a specific substance or group of related substances (such as macromolecules, small molecules, ions) into, out of, within or between cells.	-	**0.00E+00**	-
GO:0005216	ion channel activity	-	**0.00E+00**	-
GO:0007268	synaptic transmission	-	**0.00E+00**	-
GO:0019226	transmission of nerve impulse	-	**0.00E+00**	-
GO:0005261	cation channel activity	-	**0.00E+00**	-
GO:0022836	Catalysis of the transmembrane transfer of a solute by a channel that opens in response to a specific stimulus.	-	**0.00E+00**	-
GO:0022838	Catalysis of energy-independent facilitated diffusion, mediated by passage of a specific solute through a transmembrane aqueous pore or channel.	-	**0.00E+00**	-
GO:0015672	monovalent inorganic cation transport	-	1.64E-01	-
GO:0006813	potassium ion transport	-	8.96E-02	-
GO:0022832	Catalysis of the transmembrane transfer of a solute by a channel whose open state is dependent on the voltage across the membrane in which it is embedded.	-	4.24E-03	-
GO:0005267	potassium channel activity	-	1.97E-01	-
GO:0022843.	Catalysis of the transmembrane transfer of a cation by a voltage-gated channel. A cation is a positively charged ion	-	5.76E-02	-
