# Supplementary material for: Age-Correlated Gene Expression in Normal and Neurodegenerative Human Brain Tissues
Source: PLoS One. 2010 Sep 29;5(9):e13098. doi: 10.1371/journal.pone.0013098 (PMC2947518; doi:10.1371/journal.pone.0013098)
Supplement: Table S2 — (0.20 MB PDF) [file pone.0013098.s004.pdf]

Table S2.

Number of genes that show both age-correlated and disease-correlated gene expression ($p < 0.001$) in the matched regions. Age correlated genes in each region are selected using p cutoff at 0.005. The gene overlapping is done using EntrezID converted based on each microarray platform. Universe is the union of all the genes on training data set (D1 or D2) and target data set (D4 or D5).

<table>
<tr><th>Disease dataset</th><th colspan="2">AD (D5)</th><th>FTLD-TDP GRN+ (D4)</th><th>FTLD-TDP GRN- (D4)</th></tr>
<tr><td>Normal brain aging data set</td><td>BA9 (D2)</td><td>BA10 (D1)</td><td>BA10 (D1)</td><td>BA10 (D1)</td></tr>
<tr><td>Number of genes in both datasets</td><td>20271</td><td>20338</td><td colspan="2">13197</td></tr>
<tr><td>Number of age- correlated genes</td><td>202</td><td>72</td><td colspan="2">72</td></tr>
<tr><td>Number of differentially expressed genes in the disease</td><td colspan="2">735</td><td>746</td><td>182</td></tr>
<tr><td>Overlap</td><td>48</td><td>30</td><td>54</td><td>21</td></tr>
<tr><td>P-value (Fisher's exact test)</td><td>0.002</td><td>3.34×10^{-4}</td><td>2.02×10^{-6}</td><td>1.21×10^{-4}</td></tr>
</table>
