# Supplementary material for: Age-Correlated Gene Expression in Normal and Neurodegenerative Human Brain Tissues
Source: PLoS One. 2010 Sep 29;5(9):e13098. doi: 10.1371/journal.pone.0013098 (PMC2947518; doi:10.1371/journal.pone.0013098)
Supplement: Table S3 — (0.18 MB PDF) [file pone.0013098.s005.pdf]

Table S3. List of genes that show both age-correlated and disease-correlated (FTLD-U) gene expression.

Age correlated genes are selected using p cutoff at 0.01.

Gene	(1) Linear regression		(2) FTLD vs Ctrl		Description
	β_1	P-value	Log_2 Ratio	P-value	
COL4A5	↗ 0.0229	0.0069	↗ 1.59	5.25E-05	COLLAGEN, TYPE IV, ALPHA 5 (ALPORT SYNDROME)
ADD3	↗ 0.0242	0.0039	↗ 1.26	8.62E-04	ADDUCIN 3 (GAMMA)
BRSK2	↘ -0.1142	0.0057	↘ -1.01	3.51E-04	BR SERINE/THREONINE KINASE 2
SEPP1	↗ 0.0272	0.0043	↗ 1.52	1.56E-04	SELENOPROTEIN P, PLASMA, 1
RDX	↗ 0.0176	0.0074	↗ 1.09	9.82E-04	RADIXIN
ABCA8	↗ 0.0497	0.0080	↗ 2.19	1.36E-05	ATP-BINDING CASSETTE, SUB-FAMILY A (ABC1), MEMBER 8
PDE4DIP	↗ 0.0295	0.0004	↗ 1.51	8.74E-05	PHOSPHODIESTERASE 4D INTERACTING PROTEIN (MYOMEGALIN)
KCNJ4	↗ 0.0115	0.0030	↘ -1.15	2.22E-04	POTASSIUM INWARDLY-RECTIFYING CHANNEL, SUBFAMILY J, MEMBER 4
RIN2	↗ 0.0259	0.0004	↗ 1.08	7.72E-04	RAS AND RAB INTERACTOR 2
APOD	↗ 0.0234	0.0013	↗ 1.54	2.92E-07	APOLIPOPROTEIN D
HSPA2	↗ 0.0408	0.0022	↗ 1.52	1.60E-04	HEAT SHOCK 70KDA PROTEIN 2
PDE8A	↗ 0.0267	0.0053	↗ 1.21	4.78E-04	PHOSPHODIESTERASE 8A
SEP10	↗ 0.0258	0.0046	↗ 1.12	3.94E-04	SEPTIN 10
VCAN	↗ 0.0288	0.0005	↗ 1.58	7.38E-06	CHONDROITIN SULFATE PROTEOGLYCAN 2 (VERSICAN)
FRMD4B	↗ 0.0406	0.0028	↗ 1.37	3.40E-04	FERM DOMAIN CONTAINING 4B
LAMP2	↗ 0.0321	0.0028	↗ 1.05	1.45E-04	LYSOSOMAL-ASSOCIATED MEMBRANE PROTEIN 2
HSPA2	↗ 0.0282	0.0061	↗ 1.52	1.60E-04	HEAT SHOCK 70KDA PROTEIN 2
