# Supplementary material for: Age-Correlated Gene Expression in Normal and Neurodegenerative Human Brain Tissues
Source: PLoS One. 2010 Sep 29;5(9):e13098. doi: 10.1371/journal.pone.0013098 (PMC2947518; doi:10.1371/journal.pone.0013098)
Supplement: Table S4 — (0.18 MB PDF) [file pone.0013098.s006.pdf]

**Table S4**. List of genes that show both age-correlated and disease-correlated (AD) gene expression (p < 0.001). Age correlated genes in D1 are selected using p cutoff at 0.005.

| Gene | (1) Linear regression | | (2) AD vs Ctrl | | Description |
|---|---|---|---|---|---|
| | $\beta_1$ | P-value | $Log_2$ Ratio | P-value | |
| RGS4 | ↘ -0.0152 | 0.0039 | ↘ -1.64 | 7.04E-06 | REGULATOR OF G-PROTEIN SIGNALING 4 |
| SEPP1 | ↗ 0.0272 | 0.0041 | ↗ 1.52 | 5.40E-06 | SELENOPROTEIN P, PLASMA, 1 |
| SPON1 | ↗ 0.0247 | 0.0016 | ↗ 1.70 | 1.57E-06 | SPONDIN 1, EXTRACELLULAR MATRIX PROTEIN |
| EFEMP2 | ↗ 0.0083 | 0.0028 | ↗ 1.34 | 2.10E-06 | EGF-CONTAINING FIBULIN-LIKE EXTRACELLULAR MATRIX PROTEIN 2 |
| VCAN | ↗ 0.0186 | 0.0009 | ↗ 1.90 | 3.53E-07 | VERSICAN |
| LAMP2 | ↗ 0.0321 | 0.0036 | ↗ 1.21 | 6.41E-06 | LYSOSOMAL-ASSOCIATED MEMBRANE PROTEIN 2 |
| GPRC5B | ↗ 0.0188 | 0.0048 | ↗ 1.81 | 1.21E-08 | G PROTEIN-COUPLED RECEPTOR, FAMILY C, GROUP 5, MEMBER B |
| PODXL | ↗ 0.0193 | 0.0031 | ↗ 1.00 | 2.70E-06 | PODOCALYXIN-LIKE |
| CSNK1G2 | ↗ 0.0142 | 0.0045 | ↗ 1.01 | 7.45E-06 | CASEIN KINASE 1, GAMMA 2 |
