# Supplementary material for: Age-Correlated Gene Expression in Normal and Neurodegenerative Human Brain Tissues
Source: PLoS One. 2010 Sep 29;5(9):e13098. doi: 10.1371/journal.pone.0013098 (PMC2947518; doi:10.1371/journal.pone.0013098)
Supplement: Table S5 — (0.19 MB PDF) [file pone.0013098.s007.pdf]

Table S5. List of genes that show both age-correlated and disease-correlated (AD) gene expression ($p < 0.001$). Age correlated genes in D2 are selected using p cutoff at 0.005.

Gene	(1) Linear regression		(2) AD vs Ctrl		Description
	β_1	P-value	Log_2 Ratio	P-value	
GFAP	↗ 0.0226	5.88E-05	↗ 3.27	4.64E-11	GLIAL FIBRILLARY ACIDIC PROTEIN
MXI1	↗ 0.0106	0.0003	↗ 1.19	2.04E-08	MAX INTERACTOR 1
MYBPC1	↗ 0.0207	0.0019	↗ 1.47	6.33E-07	MYOSIN BINDING PROTEIN C, SLOW TYPE
ERBB2IP	↗ 0.0228	0.0030	↗ 1.48	1.11E-06	ERBB2 INTERACTING PROTEIN
AEBP1	↗ 0.0197	0.0020	↗ 2.23	1.78E-06	AE BINDING PROTEIN 1
TAX1BP3	↗ 0.0309	0.0029	↗ 1.44	3.53E-06	TAX1 (HUMAN T-CELL LEUKEMIA VIRUS TYPE I) BINDING PROTEIN 3
WWOX	↗ 0.0172	0.0029	↗ 0.97	4.87E-06	WW DOMAIN CONTAINING OXIDOREDUCTASE
KTN1	↗ 0.0143	0.0004	↗ 1.32	1.02E-05	KINECTIN 1 (KINESIN RECEPTOR)
ARHGEF6	↗ 0.0126	0.0003	↗ 0.69	1.73E-05	RAC/CDC42 GUANINE NUCLEOTIDE EXCHANGE FACTOR (GEF) 6
PSMD4	↗ 0.0098	0.0023	↘ -1.06	3.34E-06	PROTEASOME (PROSOME, MACROPAIN) 26S SUBUNIT, NON-ATPASE, 4
AP1S2	↗ 0.0122	0.0017	↘ -1.30	4.13E-06	ADAPTOR-RELATED PROTEIN COMPLEX 1, SIGMA 2 SUBUNIT
IDS	↘ -0.0259	0.0008	↗ 0.63	4.89E-06	IDURONATE 2-SULFATASE
NFRKB	↘ -0.0062	0.0034	↘ -1.50	8.15E-06	NUCLEAR FACTOR RELATED TO KAPPAB BINDING PROTEIN
DLG3	↘ -0.0205	0.0046	↘ -0.83	1.05E-05	DISCS, LARGE HOMOLOG 3 (DROSOPHILA)
CAMSAP1	↘ -0.0158	0.0028	↘ -0.92	1.17E-05	CALMODULIN REGULATED SPECTRIN-ASSOCIATED PROTEIN 1
