# Supplementary material for: Age-Correlated Gene Expression in Normal and Neurodegenerative Human Brain Tissues
Source: PLoS One. 2010 Sep 29;5(9):e13098. doi: 10.1371/journal.pone.0013098 (PMC2947518; doi:10.1371/journal.pone.0013098)
Supplement: Supplemental Methods S1 — (0.27 MB PDF) [file pone.0013098.s008.pdf]

Suppmental Methods

Estimation of the error of our age predictor   We used five-fold cross validation to estimate the error of our age predictor as follows. We first split the subjects into five cohorts. We then used reference data from four of the cohorts (training set) to generate age predictions for the individuals in the fifth cohort (test set). Thus, the average prediction error over all five cohorts is the estimated error of the age predictor:

$$\text{Prediction Error} = \left(\sum_{i=1}^{5} \sum_{j=1}^{M_i} \left| \text{Pred\_A}_{ij} - A_{ij} \right| / M_i \right) / 5 \qquad (1)$$

Here M_i is the total number of individuals in the ith partition (test set), and A_{ij} and Pred_A_{ij} are the actual and predicted ages for the jth individual in the ith partition. We computed the significance of the error by obtaining 1,000 randomized cross-validation errors with age information randomly shuffled; the significance of the prediction error is the fraction of the 1,000 randomized errors lower than the actual cross-validation error.

Calibration across different studies using housekeeping genes

One challenge that arises when performing secondary analyses of data from multiple microarray studies is the fact that different technological platforms and different experimental conditions create baseline differences between studies [1]. We therefore developed a calibration method using the expression of a set of housekeeping genes to normalize across microarray studies.

In order to apply our age predictor across diverse microarray experiments, we needed to address two issues: microarray platform differences and baseline differences attributable to

variations in experimental technique. To address the former, we used the best-match probeset ID tables provided by Affymetrix (https://www.affymetrix.com/support/technical/comparison_spreadsheets.affx (needs free NetAffx account)) to match probeset IDs on different human genome microarrays used in this paper.

For the latter, we assumed that the difference between two microarray experiments is a constant offset. We adjusted this baseline difference by estimating the difference between the expression levels of housekeeping genes common to the two datasets. 575 established housekeeping genes [2] were used in this calibration.

We describe the calibration procedure as follows:

1. Calculate the variance of all the housekeeping genes common to two datasets across healthy individuals (here we used Affymetrix Human Genome microarrays).
2. Choose housekeeping genes with variances smaller than the median of variances; calculate the median of the selected housekeeping genes for each dataset. Calculate the difference of medians of housekeeping genes on the two microarray platforms and adjust gene expression levels of target genes accordingly.

We tested the efficacy of our calibration method by evaluating the difference between median predicted age and median actual age of individuals in two datasets (datasets D1 and D4 in Table 1). We found that the difference between median predicted age and median actual age were 3.01 years and 2.99 years, and none were significant by Wilcoxon's test (Figure S2). In our analyses, the calibration made little difference implies that experimental baseline differences between microarray studies can be ignored.

1. Rhodes DR, Yu J, Shanker K, Deshpande N, Varambally R, et al. (2004) ONCOMINE: a cancer microarray database and integrated data-mining platform. Neoplasia 6: 1-6.
2. Eisenberg E, Levanon EY (2003) Human housekeeping genes are compact. Trends Genet 19: 362-365.
